# Development of a Childhood Attachment and Relational Trauma Screen (CARTS): a relational-socioecological framework for surveying attachment security and childhood trauma history

**DOI:** 10.3402/ejpt.v4i0.20232

**Published:** 2013-04-09

**Authors:** Paul A. Frewen, Barrie Evans, Jason Goodman, Aaron Halliday, James Boylan, Greg Moran, Jeffrey Reiss, Allan Schore, Ruth A. Lanius

**Affiliations:** 1 Department of Psychology, The University of Western Ontario, London, Ontario, Canada; 2 Department of Psychiatry, The University of Western Ontario, London, Ontario, Canada; 3 Graduate Program in Neuroscience, The University of Western Ontario, London, Ontario, Canada; 4 Department of Psychiatry and Biobehavioral Sciences, UCLA David Geffen School of Medicine, Los Angeles, CA, USA

**Keywords:** Child abuse and neglect, child maltreatment, trauma, attachment, family relationships, scale development

## Abstract

**Background:**

Current psychometric measures of childhood trauma history generally fail to assess the relational-socioecological context within which childhood maltreatment occurs, including the relationship of abusers to abused persons, the emotional availability of caregivers, and the respondent's own thoughts, feelings, and actions in response to maltreatment.

**Objective:**

To evaluate a computerized approach to measuring the relational-socioecological context within which childhood maltreatment occurs.

**Method:**

The psychometric properties of a Childhood Attachment and Relational Trauma Screen (CARTS) were evaluated as a retrospective survey of childhood maltreatment history designed to be appropriate for completion by adults. Participants were undergraduates (*n*=222), an internet sample (*n=*123), and psychiatric outpatients (*n*=30).

**Results:**

The internal reliability, convergent, and concurrent validity of the CARTS were supported across samples. Paired differences in means and correlations between rated item-descriptiveness to self, mothers, and fathers also accorded with findings of prior attachment and maltreatment research, illustrating the utility of assessing the occurrence and effects of maltreatment within a relational-socioecological framework.

**Conclusions:**

Results preliminarily support a new survey methodology for assessing childhood maltreatment within a relational-socioecological framework. Further psychometric evaluation of the CARTS is warranted.

Socioecology has emerged as one of the dominant meta-paradigms for understanding childhood experience and early relationships (Bronfenbrenner, 1977, 1979, 1986), including the short- and long-term effects of childhood abuse and neglect (e.g., Belsky, 1980; Cicchetti & Lynch, 1993; Cicchetti & Toth, 2005; Garbarino, 1977; Stith et al., 2009; Zielinski, & Bradshaw, 2006). Socioecological frameworks model occurrences of childhood maltreatment as intrinsically *relational* in nature. Specifically, socioecology emphasizes the fact that childhood maltreatment occurs within the context of relationships between a perpetrator(s) and a victim(s). Moreover, each persons’ thoughts, feelings, and actions are irrevocably influenced by, and in turn co-create, the greater social microsystems (e.g., families, peer relationships), exosystems (e.g., communities), and macrosystems (e.g., societies, cultures) within which each person is embedded. Unfortunately, rather than isolated occurrences, socioecological frameworks recognize that many instances of abuse and neglect occur within the context of “pathogenic relational environments” (Cicchetti & Toth, 2005, p. 409) characterized by chronic exposure to violence and abuse. Consideration of the relational-socioecological context within which maltreatment occurs is therefore paramount to any thorough account of a person's response to childhood abuse and neglect. Indeed, in the study of outcomes of other forms of abuse such as rape, relational-socioecological variables have been shown to significantly moderate psychosocial outcomes, for example, rapes perpetrated by strangers, acquaintances, or dating partners (e.g., Koss, 1985; Koss, Dinero, Seibel, & Cox, 1988).

Despite the theoretical significance of relational-socioecological frameworks to any deep understanding of a person's response to childhood maltreatment, relatively little empirical research has explicitly examined childhood maltreatment from a socioecologically informed framework, partly owing to the fact that psychometric measures of maltreatment history often fail to take sufficient account of the relational-socioecological context within which instances of childhood abuse and neglect occur. Specifically, the basic structure of many frequently used retrospective measures of childhood maltreatment history, including the Childhood Trauma Questionnaire (CTQ; Bernstein et al., 2003) and the Traumatic Antecedents Questionnaire (TAQ; Herman, Perry, & Van der Kolk, 1989), involves querying to what degree respondents endorse survey items such as “I was physically abused”. Notice, however, that persons’ answers to such questions literally tell us nothing about their relationship to the perpetrator(s), the general quality of the family environment supporting them, or their thoughts, feelings, and own actions in response to having been abused. By contrast, other frequently used maltreatment history questionnaires and interviews, including the Trauma History Questionnaire (Green, 1996; Hooper, Stockton, Krupnick, & Green, 2011), Stressful Life Events Screening Questionnaire (Goodman, Corcoran, Turner, Yuan, & Green, 1998), Traumatic Events Screening Instrument (reviewed by Ford, 2009), Computerized Assessment of Maltreatment Inventory (Dilillo et al., 2010), Juvenile Victimization Questionnaire (Finkelhor, Hamby, Ormrod, & Turner, 2005; Finkelhor, Ormrod, Turner, & Hamby, 2005), and Children's Experience of Violence Questionnaire (CEVQ; Tanaka et al., 2012; Walsh, MacMillan, Trocmé, Jamieson, & Boyle, 2008) do acquire information about perpetrators directly involved in abuse. However, these instruments generally fail to characterize other aspects of the relational-socioecological environment such as the presence versus absence of caregiver or peer support. Moreover, these instruments typically ask questions about perpetrators only within an open-text follow-up question format secondary to questioning whether maltreatment occurred at all. Consequently, the datasets rendered by such survey approaches are rarely readily amenable to detailed analysis of the outcomes of maltreatment occurring within the context of different relational-socioecological environments.

To address these problems, we undertook the task of developing a survey methodology and related data format that explicitly assessed childhood maltreatment history within a socioecological framework, here restricted to the microsystem of the family. The principal innovation of our survey methodology was to phrase items such that they referred to specific persons and then to devise a response format that simultaneously assessed the applicability of items as descriptions of all family members, including the applicability of items as descriptions of the respondent him or herself. For example, a survey item such as “This person was physically abusive” would be rated in terms of its descriptiveness of the participant's mother and father, as well as of the participant him or herself while he or she was a child. Consequently, our survey methodology queried not only whether maltreatment occurred, but in what *relational-socioecological context* (e.g., whether the respondent him or herself, and/or his or her mother and/or father were physically abusive). Notice that responses to such questions are intrinsically more informative than responses to questions about the general applicability of statements like “I was physically abused” because they specify not only what occurred (e.g., physical abuse) but also in what *relational-socioecological context* (i.e., *who* did *what*, e.g., *who* was physically abusive).

Moreover, in addition to behaviorally defined items potentially indicative of overt abuse and neglect, we included survey items to assess how generally warm, secure, and supportive the respondent considered each person in his or her family to be. For example, respondents rated for each family member whether they believed that “This person liked me” and whether “I liked this person”. Broadening the assessment of childhood maltreatment history to include general indicators of warmth, security, and support allowed us to evaluate the hypothesis that “ultimately it may be the child's perceptions of being unloved, unwanted and uncared for that count more toward their social and emotional health and adjustment than [overt] maltreatment per se” (MacKenzie, Kotch, Lee, Augsberger, & Hutto, 2011, p. 2397). Moreover, assessing the *self*-descriptiveness of items such as “I liked this person” allowed us to assess the positivity of respondents’ self-concept during their early life, potentially partly reflecting the influence of the greater family microsystem. In other words, a participant's decision not to select him or herself as a person whom he or she liked, for example, could be interpreted as a face-valid indicator of negative self-referential processing.

In summary, the primary aim of our research was to evaluate a new survey *methodology* for the assessment of childhood trauma history. We distinguish this goal from the comparably simpler task of only validating a new set of survey *items*. Indeed we envision that other item contents could also be administered within the format of the assessment procedure developed herein. Nevertheless, the scale we constructed for the present studies, collectively referring both to the survey methodology and item listing, we hereby title the Childhood Attachment and Relational Trauma Screen (CARTS). Within the scope of the present project, the CARTS was designed as a retrospective survey of childhood maltreatment history appropriate for completion by adults. The present report describes three studies that analyzed responses to the CARTS within university student (*n*=222), community (internet; *n*=127), and mental health outpatient (*n*=30) samples as an initial demonstration of the CARTS methodology for documenting childhood trauma history within a relational-socioecological framework.

Our study objectives included not only to develop and preliminarily evaluate the CARTS through standard measures of internal, convergent, and incremental concurrent validity, but further to evaluate the kinds of analyses uniquely provided for by a survey methodology that explicitly epitomizes a socioecological-relational framework. To this end, we investigated mean differences as well as correlations among item endorsements between different family members, comparing items endorsed as descriptive of respondents’ biological mothers, biological fathers, and of the respondents’ themselves. For example, comparison of mean endorsements for items such as “This person was physically abusive,” between biological mothers and fathers, facilitated investigation of established sex differences in caregiver perpetration of childhood abuse and neglect (e.g., Public Health Agency of Canada [PHAC], 2010), whereas associations between the applicability of such items as descriptions not only of the respondents’ parents but also of respondents’ themselves evaluated whether the likelihood of physically abusive behavior in the respondent him or herself was increased within the socioecological context of parental physical abuse. In comparison, differences in mean endorsements for positively framed items and those indicative of secure attachment, for example “This person helped me feel better when I was sad or upset,” made possible comparison of sex differences in parental emotional availability and support (e.g., Lum & Phares, 2005). In order to simplify such analyses of sex differences between parents, an inclusion criterion for the present study was that participants must have completed the CARTS whilst including ratings referring to both of their biological parents.

## Method

### Participants

#### Sample 1

Undergraduate university students (*n*=230) completed the CARTS for partial course credit in an introductory psychology class. Eight participants did not include a biological mother and/or father within their family list and so were excluded from further analysis (remaining *n*=222). The final student sample was mostly female (*n=*185, 85%) and of young adult age (range 17–26, *M*=18.40, *SD*=1.04). Participants completed the CARTS on private computers within groups of 12 or fewer at a campus computer laboratory in the presence of an experimenter. Sample 1 was evaluated before Samples 2 and 3; thus Sample 1 results provided hypotheses for replication and extension in other samples.

#### Sample 2

A total of 261 participants were recruited via web-links posted either on the principal investigator's university faculty home page or on other pages describing content directly pertinent to the subject of childhood maltreatment and mental health (and considered reputable as such by the corresponding author). Participants completed the CARTS through a secure website via an internet connection that was available to them. Of the 261 persons recruited, 43 did not report on both biological parents and were thus excluded from further analysis. Of the remaining 218 participants, 123 (56%) completed the survey in full and represent the sample upon which analyses are based. Completers versus non-completers were compared in their response to introductory demographic questions, for which data was available for all 218 participants. In comparison with non-completers, completers were on average five years older (*t*[216]*=*3.23, *p*=0.001) and were less likely to rate their marital status as single (28% vs. 43%), correspondingly being more likely to be either in a dating relationship (13% vs. 8%) or to be separated or divorced (14% vs. 5%). There was also a trend (*p=*0.07) for completers to be more likely to have suffered from a psychiatric condition at some time in their lives (70% vs. 43%), and a trend (*p=*0.08) for completers to more often have completed a graduate or professional degree (23% vs. 13%). Completers did not significantly differ from non-completers in gender distribution (85% vs. 79% female, *p*=0.22). The mean age of the completer sample was 37.37 (*SD*=12.49, range 18–69).

#### Sample 3

Thirty individuals (83% female, mean age=42.00, *SD*=12.66, range 18– 59) who were seeking outpatient psychological services for psychiatric problems at one of two Ontario hospitals took part in this study. All participants reported on both biological parents and so were included in the analysis. Although the present study did not include a formal diagnostic assessment, all participants reported presenting problems consistent either with a diagnosis of posttraumatic stress disorder and/or depression at the time of testing as indicated by review of medical charts at the treating hospital and/or informal assessment of presenting problems at the time of evaluation. Participants completed the CARTS on an office computer in the presence of a research assistant.

## Development of the CARTS

### Item content of the CARTS

In brief, the item listing of the CARTS was developed over an iterative process as informed by common conventions in psychometric scale development (e.g., DeVellis, 2012; Nunnally & Bernstein, 1994; Schultz & Whitney, 2005). Nevertheless, we also wish to emphasize that the current item listing, intended only as a screening measure, did *not* aim to comprehensively assess all family related variables that might be predictive of response to maltreatment (i.e., to maximize content validity). Instead, our primary aim was to develop a relatively short item-listing as a highly face-valid screening instrument of both overt instances of childhood maltreatment as well as the general warmth, security, and supportiveness of a respondents’ family relationships.

CARTS items were originally developed by the first author after reviewing items from other measures of childhood trauma history, including those referenced in the introduction (e.g., CTQ; Bernstein et al., 2003), as well as a review of items from measures of parental caregiving and attachment behavior (e.g., Lum Emotional Availability of Parents [LEAP] scale; Lum & Phares, 2005). The concept of “outgoing” versus “incoming” feelings (e.g., “I liked this person” vs. “This person liked me”, respectively), as utilized within the Bene-Anthony Family Relations Test (Anthony & Bene, 1957; Bene & Anthony, 1957; Griffin, 2005; Parkin, 2001), was also incorporated into the structure of item design, as was coverage of the following three simple negative affective states: sad–upset, scared–worry, mad–angry. An initial set of CARTS items was presented to outpatients, clinical staff, and clerical staff at an outpatient trauma-treatment center for feedback concerning their face and content validity. Items were then rephrased, and additional items were included, based on consultation with target groups (Vogt, King, & King, 2004). We consider the resulting set of 56 CARTS items that we developed to be highly face valid and relatively straightforward in their interpretation, averaging grade 2.8 on the Flesch-Kincaid index, or a North American reading age equivalent of 8–9 years, as determined by the proofing utility within MS Word.

The full set of 56 CARTS items, rationally defined subscale structure, and additional suggestions for item interpretation are contained within Table 1. Thirteen positively framed relational descriptions were included and titled simply as a *Positive* subscale (e.g., “I liked this person very much”, “This person liked me very much”). Eight items were intended to reflect the theoretical construct of “Secure Attachment” as indicated both by “proximity seeking” behaviors (4 items, e.g., “I went to this person when I was feeling sad or upset”) and “emotional availability” behaviors (4 items, e.g., “This person helped me feel better when I was feeling sad or upset”). Three items were developed to assess “negative affective traits” (e.g., “This person was sad and upset a lot of the time”) whereas a single item was used to screen for the presence of a more positive affective disposition (i.e., “This person was usually happy”). Items were also included in order to assess negative feelings in the respondent attributed to other family members (4 items, e.g., “This person made me feel sad or upset”), and negative relational beliefs either attributed to other family members (5 items, e.g., “I thought that this person didn't love me”) or directed toward other family members (5 items, e.g., “I did not love this person”). Two item, behaviorally defined subscales were developed in order to screen for emotionally and physically abusive behavior, distinguishing between behaviors explicitly directed at the respondent him or herself (i.e., self-referential items, e.g., “This person called me bad names”, “This person slapped, smacked, or hit me”, respectively), and behaviors directed at other family members generally (i.e., non-self-referential items, e.g., “This person called people in my family bad names”, “This person slapped, smacked, or hit people in my family”, respectively). Finally, six items screened for occurrences of “Sexually Abusive” behavior directed toward the respondent (e.g., “This person made me do things to them without their clothes on”), and three additional items were phrased so as to assess what we assume to be abusive events in a less behaviorally explicit way (i.e., subscale labeled “Bad Things” in Table 1, e.g., “This person did bad things to me that I didn't like to talk about or think of”).

**Table 1:** Item listing of the Childhood Attachment and Relational Trauma Screen (CARTS)

	Item	Scale	Self-Rating Scored	Self-Refer.	Direction
1	I liked this person very much.	Positive	Yes	Yes	S-O
2	I loved this person very much.	Positive	Yes	Yes	S-O
3	This person liked me very much.	Positive	No	Yes	O-S
4	This person loved me very much.	Positive	No	Yes	O-S
5	This person took care of me.	Positive	No	Yes	O-S
6	This person cared about me.	Positive	No	Yes	O-S
7	This person was proud of me.	Positive	No	Yes	O-S
8	This person gave me hugs and kisses.	Positive	No	Yes	O-S
9	This person made me feel calm.	Positive	No	Yes	O-S
10	This person made me feel happy.	Positive	No	Yes	O-S
11	This person made me feel good about myself.	Positive	No	Yes	O-S
12	I had a lot of fun being with this person.	Positive	No	Yes	S-O ∣ O-S
13	I was happy that this person was in our family.	Positive	Yes	Yes	S-O
14	I went to this person when I was feeling sad or upset.	Secure	No	Yes	S-O
15	I went to this person when I was feeling scared or worried.	Secure	No	Yes	S-O
16	I went to this person when I was feeling mad and angry.	Secure	No	Yes	S-O
17	I went to this person for help when I had a problem.	Secure	No	Yes	S-O
18	This person helped me feel better when I was sad or upset.	Secure	No	Yes	O-S
19	This person helped me feel better when I was scared or worried.	Secure	No	Yes	O-S
20	This person helped me feel better when I was mad and angry.	Secure	No	Yes	O-S
21	This person helped me when I had a problem.	Secure	No	Yes	O-S
22	This person was sad or upset a lot of the time.	Neg. Affect	Yes	No	–
23	This person was mad and angry a lot of the time.	Neg. Affect	Yes	No	–
24	This person was scared or worried a lot of the time.	Neg. Affect	Yes	No	–
25	This person was usually happy.	Pos. Affect	Yes	No	–
26	This person made me feel sad or upset.	Neg. Feel. From	No	Yes	O-S
27	This person made me feel scared or worried.	Neg. Feel. From	No	Yes	O-S
28	This person made me feel mad and angry.	Neg. Feel. From	No	Yes	O-S
29	This person made me feel bad about myself.	Neg. Feel. From	No	Yes	O-S
30	This person called me bad names.	Emot. Abuse - Self	No	Yes	O-S
31	This person said very mean things to me.	Emot. Abuse - Self	No	Yes	O-S
32	This person called people in my family bad names.	Emot. Abuse - Other	No	No	–
33	This person said very mean things to people in my family.	Emot. Abuse - Other	No	No	–
34	I thought that this person didn't like me very much.	Neg. Beliefs From	No	Yes	O-S
35	I thought that this person didn't love me very much.	Neg. Beliefs From	No	Yes	O-S
36	I thought that this person wished that I was NOT in our family.	Neg. Beliefs From	No	Yes	O-S
37	I thought that this person thought I'm bad.	Neg. Beliefs From	No	Yes	O-S
38	I thought that this person hated me.	Neg. Beliefs From	No	Yes	O-S
39	I did NOT like this person very much.	Neg. Beliefs To	No	Yes	S-O
40	I did NOT love this person very much.	Neg. Beliefs To	No	Yes	S-O
41	I wished that this person was NOT in our family.	Neg. Beliefs To	No	Yes	S-O
42	I thought that this person was a bad person.	Neg. Beliefs To	No	Yes	S-O
43	I thought that I hated this person.	Neg. Beliefs To	No	Yes	S-O
44	This person slapped, smacked, or hit me.	Phys. Ab. - Self	No	Yes	O-S
45	This person punched or kicked me.	Phys. Ab. - Self	No	Yes	O-S
46	This person slapped, smacked, or hit people in my family.	Phys. Ab. - Other	Yes	No	–
47	This person punched or kicked people in my family.	Phys. Ab. - Other	Yes	No	–
48	This person did bad things to me that I was not supposed to tell other people about.	Bad Things	No	Yes	O-S
49	This person made me do bad things that I was not supposed to tell other people about.	Bad Things	No	Yes	O-S
50	This person did bad things to me that I didn't like to talk about or think of.	Bad Things	No	Yes	O-S
51	This person made me touch their body in places where I didn't want to.	Sexual Abuse	No	Yes	O-S
52	This person touched my body in places where I didn't want them to.	Sexual Abuse	No	Yes	O-S
53	This person made me touch their body in places where they shouldn't.	Sexual Abuse	No	Yes	O-S
54	This person touched my body in places where they shouldn't touch me.	Sexual Abuse	No	Yes	O-S
55	This person made me do things to them without their clothes on.	Sexual Abuse	No	Yes	O-S
56	This person made me do things to them without my clothes on.	Sexual Abuse	No	Yes	O-S

Notes: Self-refer.=Self-referential item. S-O=feeling/thought/behavior originating in the self (respondent) that is directed at another person. O-S=feeling/thought/behavior perceived to be directed at the respondent as originating within another person.

It is important to note that the self-referential phrasing chosen for many of the CARTS items tended to make them intrinsically less applicable to self-endorsement than as a description of other persons within the family. For example, referring to CARTS *emotionally* and *physically abusive* items, although the self might be endorsed as among people who “called people in my family bad names” and/or “slapped, smacked, or hit people in my family”, for most participants it will be less logical to consider the self as a person who potentially “called me bad names” or “slapped, smacked, or hit me”. For this reason, although available as a potential response option for all items, self-ratings were scored and submitted to statistical analysis only for non-self-referential items, in addition to three items from the *positive* subscale that were considered to be general enough in phrasing that they might be considered relevant to self-endorsement. The latter items were treated as a screening indicator of positive appraisals of oneself generally as a person, as well as specifically within the context of one's membership within the family. Table 1 indicates the items scored for self-ratings in the present studies.

### Computerized administration of the CARTS

**Fig. 1:**
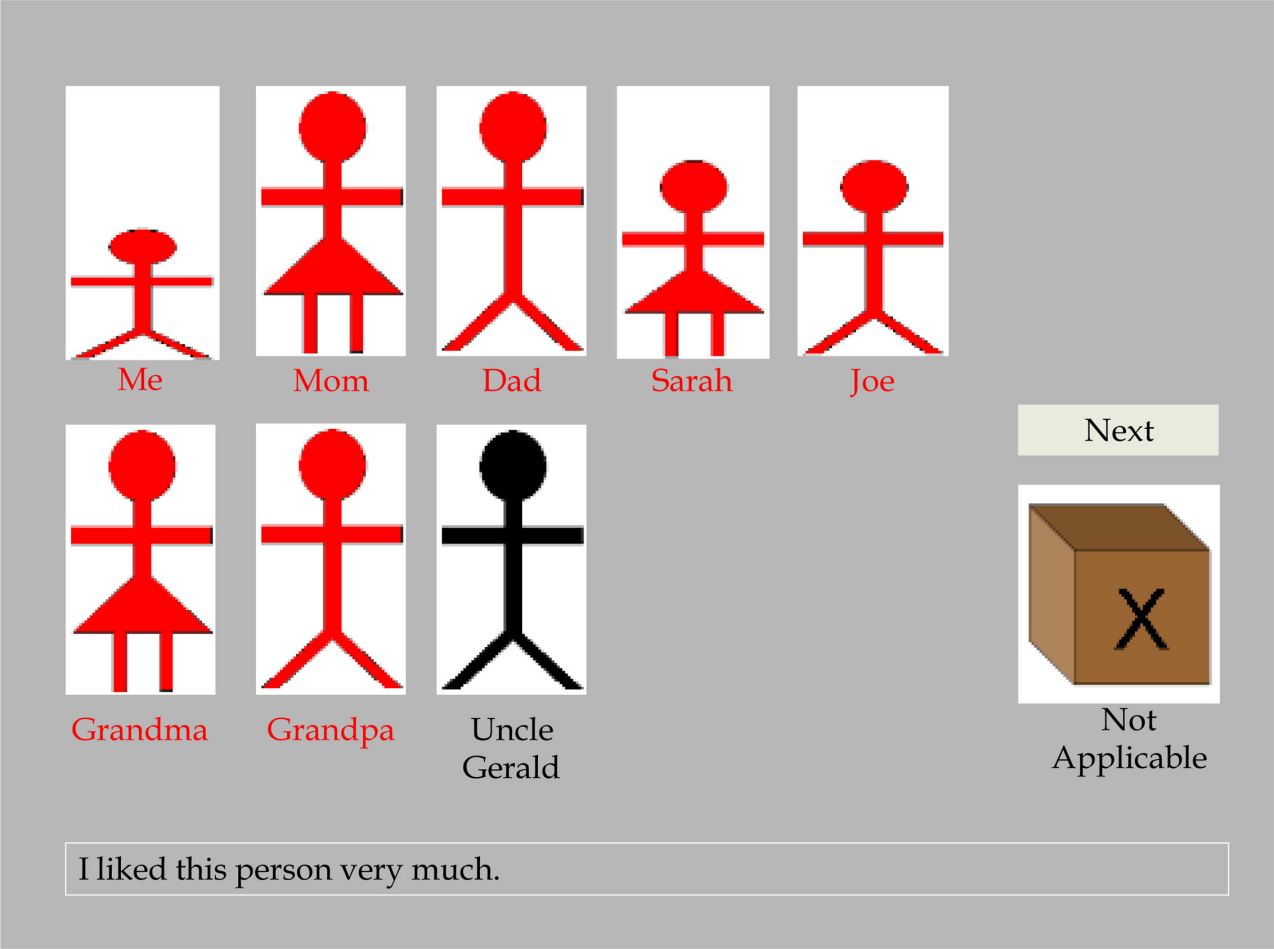
*Illustration of the CARTS survey methodology*. In this example, a respondent has been presented with the test item “I liked this person very much”, and each of the figures and labels would have initially been shown in black ink. That the majority of the figures and labels are presently in red ink illustrates that the respondent has indicated, by clicking on the following respective figures/labels that, when growing up as a child and adolescent, he liked himself, both of his parents, his older siblings (sister “Sarah” and brother “Joe”), and his grandparents (all denoted in red). However, the respondent has indicated, in omitting clicking “Uncle Gerald” (still denoted black), that he did not like his uncle very much. Should the respondent have wished to indicate that he did not like *any* of these persons, including her/himself, she/he would have clicked the brown box marked by an “X” and labeled “Not Applicable”. Clicking the “Next” button would occasion the presentation of a new test item, with all figures and labels returning to the default black ink. Different types of items were presented. For example, presented with an item indicative of “Physically Abusive” behavior (e.g., “This person slapped, smacked, or hit me”), the participant might have clicked on a different set of individuals, or indicating that “Physically Abusive” behavior had not occurred at all during his/her childhood by clicking “Not Applicable”.

Please see Fig. 1 for an illustration of how respondents complete the CARTS. Administration of the CARTS was fully automated by computer. Participants were first instructed to: “Please type in the names of up to 11 people who were in your family *when you were growing up (as a child and/or a teenager)*. Then click in the list beside to indicate their relationship to you. Please feel free to define “family” however you wish; for example, whether you choose to include extended family and friends is entirely up to you, just remember that you will only be able to include up to a maximum of 11 people. Unfortunately, pets can't be included in the survey.” Participants then typed in up to 11 family members and, for each, selected a label from a drop-down menu that defined their relationship to each person. Selection options were extensive and explicitly assessed the biological relationship of the respondent to each family member (e.g., “Biological Mother” versus “Non-Biological Mother [e.g., adoptive, step-mother, etc.]”). As previously noted, to be included in the present set of analyses, participants must have reported on both their biological parents. Nevertheless, the instructions given to participants allowed them to define “family” as liberally as they wished such that extended family (e.g., grandparents, uncles, aunts, etc.), friends, and others (e.g., teachers, etc.) could be included, and specific family members (e.g., biological parents) could be *excluded*, entirely at the respondents’ discretion. This was intended so as to collect a fully unbiased, idiographic, participant-specified characterization of the respondents’ socioecological environment at the familial microsystem level. Notably pets (animals), however, could not be included in the listing. The CARTS computer program saved a basic visual representation (a “stick-figure”-like icon) of each relationship rated, characterized only by: 1) the gender of the relationship to be rated, and 2) three sizes intended to differentiate between an adult, a child older than the participant, and a child younger or of similar age to the participant. This icon was presented above a label indicating the referent it denoted (e.g., “Me”, “Mom”, “Dad”).

Participants were then instructed: “You will now be presented with a number of statements. Please read each statement and *click on the people that the statement was true for when you were growing up (as a child and a teenager)*. If a person is clicked, his or her picture and name will turn red, indicating that they are selected. If you change your mind, click again and their picture and name will turn back to black, indicating that they are not selected. Click “Me” if the statement describes *your own feelings, thoughts, and/or behavior when you were growing up (as a child and a teenager)*. If the statement was not true for *anyone*, click the brown box labeled “Not Applicable.” When all of the people have been included for a particular statement, click the “Next” button. Click “Begin” to start”. Upon clicking a “Begin” button, participants were presented each survey item one at a time, and indicated to which family member(s) the item applied as a description at the time the respondent “was growing up (as a child and a teenager)” (time period intended to be directly consistent with that employed for the CTQ; Bernstein et al., 2003). Participants made their ratings by clicking with their computer mouse on the associated “stick-figure” icons or labels (see exemplar in Fig. 1). As per the instructions given, any combination of persons could be selected, and participants could revise their answers by clicking on the names or icons more than once. Participants indicated that an item did not apply to *anyone* in their family by clicking on a box marked by an “X” and denoted “Not Applicable”. It is important to note, however, that by the current procedure it was not possible to indicate that an item(s) applied as a description of persons other than the family members the participant had previously listed.

Participants’ responses were saved as a tally of which items were attributed to which individuals including the self (i.e., scored as 1=selected, 0=not selected). An overall rendering of the *non*-applicability of any item *across* all family members was also saved (i.e., the number of items for which participants clicked the “Not Applicable” box; see Fig. 1). Completion of the CARTS typically required 20–25 minutes. Participants typically consider the procedure entirely straightforward; no participant we have so far tested in person has required any significant assistance in order to complete it.

### Statistical analysis of the CARTS

To provide a hypothesis-driven rather than exploratory approach to our initial psychometric studies of the CARTS, we analyzed only four classes of response available for all participants as a study inclusion criterion: those items that were considered applicable specifically as a description of: (1) the survey respondent him/herself; (2) the respondent's biological mother; (3) the respondent's biological father, and (4) items considered not applicable to *anyone* in the respondent's family (i.e., for which participants clicked the “Not Applicable” box; see Fig. 1). It is important to point out that the latter overall “Non-Applicability” ratings refer not only to the non-applicability of Self, Mother, and Father ratings, but additionally to all other family members that the respondent may have rated (e.g., siblings, extended family, etc.). Future studies may compare how other types of family members tend to be rated and whether this varies by the gender, age, and genetic relatedness of the family member relative to the respondent.

A first step toward validating the survey methodology of the CARTS was to assess whether the items themselves were internally consistent; recognizing the dichotomous (true–false) nature of item-level responses to the CARTS, the *Kudar-Richardson-20* statistic was calculated for all subscales as specifically referring to Not Applicable, Self, Mother and Father ratings. The frequency distributions of CARTS subscales were also examined, although violations of normality were expected. Specifically, subscales calculated on the basis of the sum of two or fewer dichotomous items by definition cannot be normal, and subscales composed of five or fewer dichotomous items are infrequently normal unless the test items are clearly graded in severity. Moreover, endorsement of maltreatment histories and significant negative affect was expected to be less frequent than lack or “partial” endorsement of such histories, such that items assessing maltreatment and significant negative affect were expected to exhibit leftward skews for person ratings, and associated positive skews for “Not Applicable” ratings, the opposite being true of items assessing positively framed items.

The convergent validity of the CARTS was evaluated in relation to the CTQ (Bernstein et al., 2003) and the LEAP (Lum & Phares, 2005), with the incremental convergent validity of CARTS parental ratings evaluated relative to CARTS Not Applicable ratings alone. In addition, the incremental concurrent validity of the CARTS was also evaluated, relative to CTQ measures of emotional, physical, and sexual abuse, in relation to measures of depression, anxiety, and stress symptoms (i.e., the 21-item version of the Depression, Anxiety, and Stress Scales [DASS-21; Lovibond & Lovibond, 1995], assessed within Sample 1), and trait positive and negative affect (i.e., the International Version of the Positive and Negative Affect Schedule [I-PANAS-SF; Thompson, 2007], assessed within Sample 2). These tests evaluated the hypothesis that CARTS measures of negative feelings and beliefs concerning the self, attributed to respondents’ biological parents, would be predictive of negative affective outcomes above and beyond explicit knowledge of abuse histories (MacKenzie et al., 2011). Finally, a short-form of the Marlowe-Crown Social Desirability Scale (Reynolds & Gerbasi, 1982) was administered to a subgroup of Sample 1 participants in order to assess whether CARTS ratings were associated with a tendency toward positive or negative impression management.

**Fig. 2:**
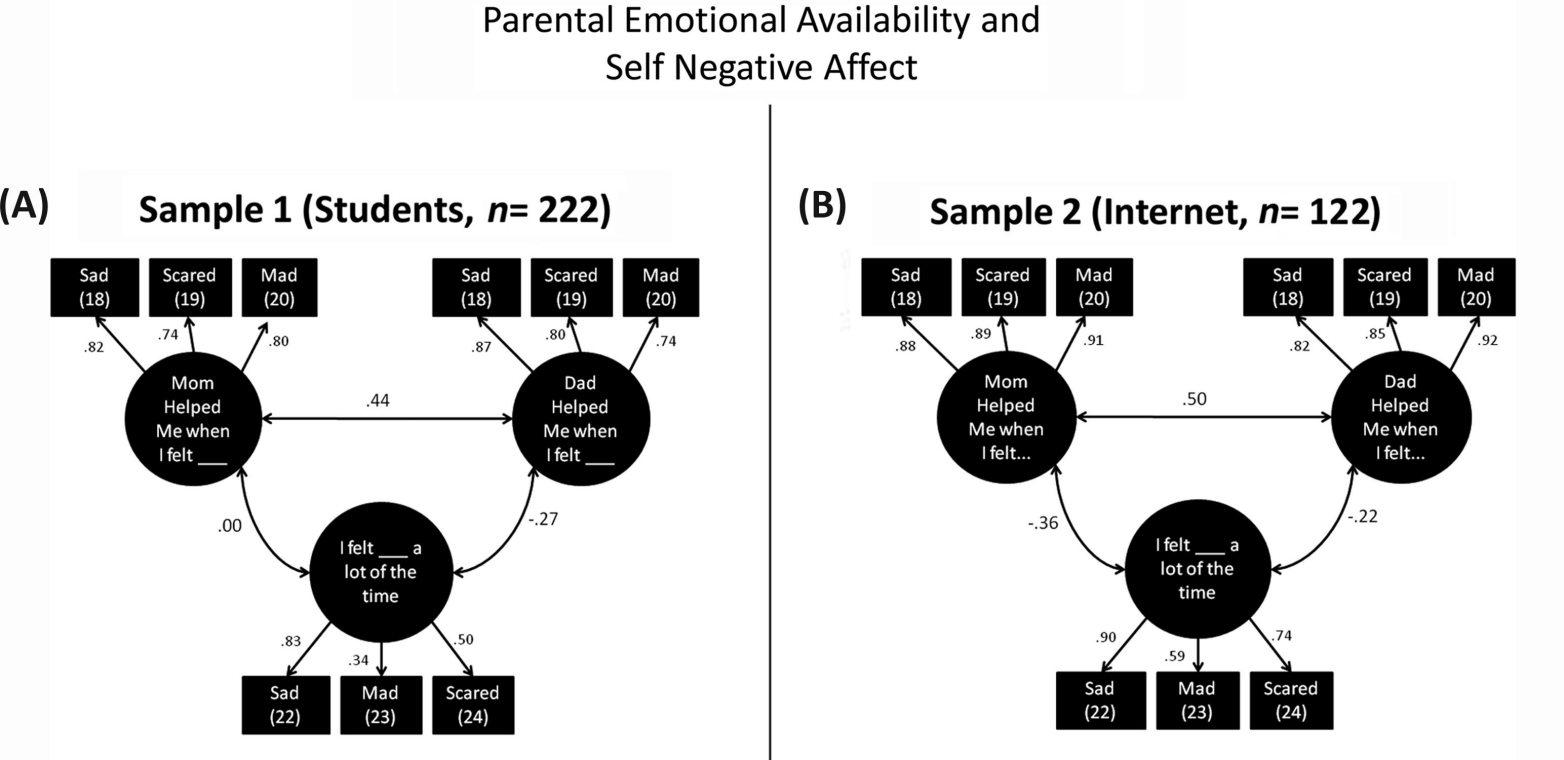
Structural equation model associating CARTS parental emotional availability with self-rated negative affect. *Note:* Errors not shown; errors for identical items between mother and father ratings (e.g., Mom-Sad, Dad-Sad) were permitted to correlate (not shown). Sample 1: Chi-square (21)=11.06, *p*=0.96. CFI>0.999. RMSEA<0.001 (PCLOSE>0.99). Sample 2: Chi-square (21)=20.92, *p*=0.46. CFI>0.999. RMSEA<0.001 (PCLOSE=0.78). Item numbers in brackets (see Table 1).

Beyond straightforward analyses of internal, convergent, incremental, and concurrent validity, however, the primary objective of the present study related to the conduct of analyses uniquely made possible by collecting survey responses within a relational-socioecological context framework. Specifically, these included: 1) paired comparisons of endorsement rates between different family members for similar items (e.g., differences between endorsement rates for CARTS *physically abusive* items as referring to mothers vs. fathers), and 2) correlations between item endorsement rates for similar items between different family members (e.g., correlations between endorsement rates for CARTS *physically abusive* items between mothers vs. fathers). Paired comparisons of means were conducted using *t*-tests and paired correlations were examined using the *τ-b* coefficient which is appropriate for ordinal-scaled measurements. In addition, as a demonstration of the more complex associations that can also be tested within socioecological-relational contextualized datasets, as rendered by the CARTS, we evaluated the fit of a structural equation model (SEM) predicting associations between CARTS low parental *emotional availability* and increased CARTS self-rated *negative affective traits* within the two larger samples, that is, Samples 1 and 2 (see Fig. 2A and 2B, respectively). Both models employed maximum likelihood estimation and fit was considered acceptable with RMSEA<0.06 and CFI>0.95 in accordance with common SEM convention (e.g., Brown, 2006; Hu & Bentler, 1999).

### Materials and procedure

Participants tested in person were seated in front of a computer at an introductory CARTS screen after providing verbal and written consent to participate in the study, whereas participants tested via the internet indicated their consent to participate after reading a letter of information on their computer screen that was presented to them after clicking a web link that advertised the study. Participants completed the CARTS in addition to a number of standard paper-and-pencil or computerized surveys. Results referring to the Depression, Anxiety, Stress Scales (within Sample 1) and an International Version of the Positive and Negative Affect Schedule (within Sample 2) will be reported on herein. These scales evidenced excellent reliability and validity in the current and in many previous studies. Whether the CARTS was completed before or after these other measures was counter-balanced across participants. The full study procedure typically required 60–90 minutes to complete for undergraduates, and 30–40 minutes for the internet and mental health outpatients samples, with these differences due to undergraduates being asked to complete a greater number of additional questionnaires that will not be the subject of analyses presented herein.

## Results

### Sample description: Childhood Trauma Questionnaire and Emotional Availability of Parents

#### Sample 1

There were relatively few reports of emotional, physical, or sexual abuse on the CTQ among undergraduates. Relative to CTQ student norms published for a representative sample of Canadian undergraduates by Paivio and Cramer (2004; *n=*433), the present student sample scored at the 37th percentile for Emotional Neglect (*M*=7.91, *SD*=3.44), 44th percentile for Emotional Abuse (*M*=7.99, *SD*=3.48), 43rd percentile for Physical Abuse (*M*=5.92, *SD*=2.29), and 42nd percentile for Sexual Abuse (*M*=5.34, *SD*=1.50). Considering the 6-point rating scale for the LEAP, on average, means for the LEAP scale indicated students rated their mothers as being “very often” (*M*=78.49, *SD*=13.37) emotionally available to them during their childhood and adolescence, whereas fathers were rated as being between “Often” and “Very Often” (*M*=68.52, *SD*=19.19) emotionally available to their children, *d’*=0.60, *t*(219)=8.76, *p*<0.001; these differences are consistent with prior results with the LEAP scale (Lum & Phares, 2005).

#### Sample 2

The majority of those participating via the internet indicated that they either currently (*n*=70, 57%) or have previously (*n*=16, 13%) “suffered from a psychiatric condition that was diagnosed by a physician or psychologist”. According to CTQ screening items (Thombs, Bernstein, Ziegelstein, Bennett, & Walker, 2007), 54% (*n*=67) reported being physically abused (“When I was growing up, people in my family hit me so hard that it left me with bruises or marks”) and 53% (*n*=65) reported being sexually abused (“When I was growing up, someone tried to touch me in a sexual way, or tried to make me touch them”). In addition, 84% (*n*=103) answered in the affirmative that they “believe that [they were] emotionally abused” during childhood.

#### Sample 3

Relative to norms published within the CTQ manual (Bernstein & Fink, 1998), means for the CTQ subscales in the mental health outpatient sample also suggested a high prevalence of histories of childhood abuse and neglect: Emotional Neglect (*M*=15.30, *SD*=5.62, 83rd percentile), Emotional Abuse (*M*=15.00, *SD*=6.59, 89th percentile), Physical Abuse (*M*=9.50, *SD*=5.28, 78th percentile), and Sexual Abuse (*M*=12.65, *SD*=7.49, 92nd percentile). Considering the 6-point rating scale for the LEAP, on average, means for the LEAP scale indicated that participants rated both their mothers (*M=*50.64, *SD=*23.30) and fathers (*M=*46.72, *SD=*23.16) as only being “sometimes” emotionally available to them during their childhood.

### Preliminary validation of the CARTS item content

#### Internal consistency and non-normality of the CARTS Self, Mother, and Father ratings

The obtained Kudar-Richardson-20 coefficients for the CARTS subscales, examined as specific to ratings for 1) “Not Applicable” altogether, 2) Self, 3) Mother, and 4) Father, are reported in Tables 2–4 for Samples 1–3, respectively. Considering the small number of items included within each subscale, internal consistency was determined to be within acceptable limits for most subscales across rating types and samples. Across all samples, the internal consistency among the three negative affective trait items was somewhat low, presumably indicating the specificity of the three different emotional states described (e.g., a family member may have been considered “sad and upset a lot of the time” but not “mad and angry …” or “scared or worried a lot of the time”). We nevertheless retained the sum score across these three items in subsequent analyses for the sake of parsimony. Referring to *physically abusive* items, internal consistency was higher for father ratings than for mother- or self-ratings, although was generally low across all rating-types analyzed. Follow-up analysis of item-level responses demonstrated that this finding was largely attributable to low endorsement of persons having “punched or kicked” the respondent and/or other family members, even if the family member was reported to have “slapped, smacked, or hit” people within the family. Nevertheless, no report of a family member having “punched or kicked” another family member(s) was made without additional endorsement of that member having “slapped, smacked, or hit” another family member(s). In this case, again for the sake of parsimony, we chose to retain the sum score across physically abusive items based on the assumption that the item describing a person “punching and kicking” others could be interpreted simply as a more severe example of physically abusive behavior than the one describing a person “slapping, smacking, or hitting” others. Finally, the internal consistency of mother ratings was lower than that observed for father ratings for items describing “bad things” as having taken place. Item-level analyses demonstrated that this effect was due to particularly infrequent endorsement among respondents as having had mothers who “made [them] do bad things that [they were] not supposed to tell other people about”. Again, for the sake of parsimony and to facilitate comparisons between mothers and fathers, the full subscale was retained.

**Table 2:** Descriptive statistics and paired comparisons between CARTS subscale ratings for “Not Applicable”, Self, Mother, and Father in the Student Sample (*n*=222)

*Subscale (No. of items)*	*Not Applicable*			*Self*			*Mother*			*Father*			*Correlations*		
				
	*α*	*M*	*SD*	*α*	*M*	*SD*	*α*	*M*	*SD*	*α*	*M*	*SD*	*τ*_bc_	*τ*_bd_	*τ*_cd_
Positive (13, 3)	0.89	0.21	1.08	0.71	0.87	1.08	0.91	10.89*	3.17	0.93	9.37	4.17	0.23*	0.28*	0.56*
Secure (8)	0.93	0.41	1.44	–	–	–	0.91	6.18*	2.61	0.90	4.18	3.05	–	–	0.36*
P-Affect (1)	–	0.08	0.27	–	0.35	0.48	–	0.61	0.49	–	0.56	0.50	0.35*	0.33*	0.62*
N-Affect (3)	0.75	1.66	1.22	0.56	0.17	0.50	0.53	0.40	0.73	0.53	0.38	0.71	0.28*	0.27*	0.25*
N-Feelings From (4)	0.83	2.07	1.61	–	–	–	0.81	0.69	1.19	0.82	1.03*	1.40	–	–	0.40*
N-Beliefs From (5)	0.85	3.82	1.67	–	–	–	0.74	0.16	0.61	0.81	0.40*	1.02	–	–	0.47*
N-Beliefs To (5)	0.88	3.92	1.69	–	–	–	0.76	0.09	0.46	0.89	0.32*	1.02	–	–	0.16
E-Ab to Self (2)	0.59	1.32	0.77	–	–	–	0.98	0.11	0.46	0.98	0.21	0.60	–	–	0.29*
E-Ab to Others (2)	0.75	1.36	0.83	0.92	0.06	0.34	0.95	0.16	0.53	0.92	0.35*	0.73	0.20*	0.10	0.47*
P-Ab to Self (2)	0.66	1.43	0.77	–	–	–	0.17	0.19	0.42	0.43	0.23	0.49	–	–	0.57*
P-Ab to Others (2)	0.74	1.58	0.72	0.92	0.03	0.24	0.40	0.11	0.35	0.70	0.19*	0.52	0.08	0.14	0.48*
Bad Things (3)	0.85	2.74	0.75	–	–	–	0.37	0.03	0.19	0.79	0.08	0.41	–	–	0.40*
S-Ab (6)	0.87	5.86	0.75	–	–	–	–	0.00	0.00	0.72	0.02	0.21	–	–	–

* *p<*0.01, two-tailed.

**Table 3:** Descriptive statistics and paired comparisons between CARTS subscale ratings for “Not Applicable”, Self, Mother, and Father in the Internet Sample (*n=*123)

*Subscale (No. of items)*	*Not Applicable*			*Self*			*Mother*			*Father*			*Correlations*		
				
	*α*	*M*	*SD*	*α*	*M*	*SD*	*α*	*M*	*SD*	*α*	*M*	*SD*	*τ*_bc_	*τ*_bd_	*τ*_cd_
Positive (13, 3)	0.90	2.50	3.34	0.74	0.42	0.84	0.94	6.28**	4.78	0.93	4.77	4.56	0.37**	0.35**	0.45**
Secure (8)	0.92	3.09	3.10	–	–	–	0.94	2.67**	3.16	0.92	1.18	2.28	–	–	0.49**
P-Affect (1)	–	0.22	0.42	–	0.07	0.25	–	0.24	0.43	–	0.24	0.43	0.24**	0.32**	0.41**
N-Affect (3)	0.80	0.59	1.01	0.79	0.89	1.13	0.57	1.33**	1.09	0.55	0.80	0.91	0.09	0.11	0.22**
N-Feelings From (4)	0.89	0.69	1.32	–	–	–	0.84	1.91	1.62	0.83	2.28*	1.61	–	–	0.26**
N-Beliefs From (5)	0.87	1.65	1.90	–	–	–	0.89	1.89	2.02	0.85	1.85	1.90	–	–	0.40**
N-Beliefs To (5)	0.86	1.83	1.93	–	–	–	0.90	1.04	1.71	0.88	1.41*	1.86	–	–	0.27**
E-Ab to Self (2)	0.74	0.60	0.81	–	–	–	0.92	0.82	0.95	0.90	0.82	0.94	–	–	0.15*
E-Ab to Others (2)	0.66	0.76	0.83	0.94	0.15	0.51	0.84	0.51	0.81	0.91	0.85**	0.95	0.13	0.08	0.15*
P-Ab to Self (2)	0.58	0.78	0.77	–	–	–	0.48	0.66	0.71	0.68	0.73	0.81	–	–	0.29**
P-Ab to Others (2)	0.73	0.96	0.86	0.56	0.04	0.24	0.61	0.48	0.69	0.75	0.70**	0.84	0.11	−0.05	0.37**
Bad Things (3)	0.91	1.72	1.36	–	–	–	0.76	0.33	0.75	0.91	0.67**	1.15	–	–	0.47**
S-Ab (6)	0.97	4.43	2.43	–	–	–	0.72	0.07	0.40	0.97	0.72**	1.82	–	–	0.13

* *p<*0.05, one-tailed (as replication of Sample 1 results)

** *p<*0.01, two-tailed (as replication of Sample 1 results and/or as new finding in Sample 2).

As hypothesized, the Kolmogorov–Smirnov and Shapiro–Wilk statistics suggested that the frequency distributions of all CARTS subscales deviated significantly from the normal distribution across all three samples (all *p*'s<0.001). In no case could this result be attributable to outliers. Instead, as expected, self, mother, and father ratings for negative-framed items, including those indicative of abusive behavior, exhibited strong leftward skews favoring non-endorsement, in turn associated with a strong positive-skew for “Not Applicable” ratings. The reverse was generally true for positive-framed items (positive and secure subscales), with the exception that father-rated positive-framed items also exhibited a leftward skew within the internet and outpatient samples, indicating many respondents did not regard their fathers as “positive” or as a basis for “secure attachment”. We did not attempt to transform CARTS data in order to render it normal prior to further data analysis.

**Table 4:** Descriptive statistics and paired comparisons between CARTS subscale ratings for “Not Applicable”, Self, Mother, and Father in the Outpatient Sample (*n*=30)

	*Not Applicable*			*Self*			*Mother*			*Father*			*Correlations*		
					
*Subscale (No. of items)*	*α*	*M*	*SD*	*α*	*M*	*SD*	*α*	*M*	*SD*	*α*	*M*	*SD*	*τ*_bc_	*τ*_bd_	*τ*_cd_
Positive (13, 3)	0.62	0.87	1.33	0.84	0.43	0.94	0.92	7.83*	4.35	0.95	5.77	5.04	0.12	0.26*	0.42**
Secure (8)	0.91	2.00	2.74	–	–	–	0.94	3.67**	3.33	0.92	2.03	2.82	–	–	0.48**
P-Affect (1)	–	0.13	0.34	–	0.17	0.38	–	0.33	0.48	–	0.40	0.50	0.63**	0.55**	0.43**
N-Affect (3)	0.89	0.83	1.23	0.73	1.03	1.07	0.52	1.13	1.04	0.41	0.83	0.83	0.28*	0.12	0.39**
N-Feelings From (4)	0.86	0.77	1.33	–	–	–	0.78	1.53	1.53	0.91	2.33*	1.77	–	–	0.20
N-Beliefs From (5)	0.89	1.70	1.99	–	–	–	0.87	1.23	1.77	0.88	1.70	1.97	–	–	0.26*
N-Beliefs To (5)	0.92	2.17	2.15	–	–	–	0.78	0.70	1.26	0.89	1.73**	2.00	–	–	0.30**
E-Ab to Self (2)	0.80	0.77	0.90	–	–	–	0.92	0.67	0.92	0.93	1.00	0.98	–	–	0.10
E-Ab to Others (2)	0.70	0.83	0.87	n.c.	0.03	0.18	0.96	0.63	0.93	0.82	0.77	0.90	−0.13	0.08	0.24
P-Ab to Self (2)	0.68	0.77	0.82	–	–	–	0.51	0.53	0.68	0..69	0.67	0.80	–	–	0.16
P-Ab to Others (2)	0.83	1.10	0.92	0.79	0.10	0.40	0.65	0.27	0.58	0.80	0.63*	0.85	−0.13	−0.02	0.34*
Bad Things (3)	0.89	1.73	1.34	–	–	–	0.59	0.20	0.55	0.87	1.00**	1.26	–	–	0.23
S-Ab (6)	0.99	4.13	2.3	–	–	–	n.c.	0.00	0.00	0.99	1.13*	2.33	–	–	–

* *p<*0.05, one-tailed (as replication of Sample 1 results)

** *p<*0.01, one-tailed (as replication of Sample 1 and/or Sample 2 results).

#### Convergent and concurrent criterion-related validity of the CARTS

Tables 5 and 6 report the results of multiple regressions evaluating the convergent and concurrent criterion-related validity of the CARTS. Regarding convergent validity, within Samples 1 and 2, CARTS ratings accounted for between 26% and 51% of the variance in CTQ subscale or item-screening scores (Table 5), and between 34% and 40% of the variance in LEAP-mother and LEAP-father ratings within Sample 1 (Table 6). In all cases excepting convergence with CTQ Sexual Abuse scores within Sample 2, inclusion of CARTS parental ratings incrementally predicted additional variance in CTQ scores beyond CARTS general “non-applicable” ratings alone. Interestingly, within the student sample, CTQ sexual abuse ratings were concurrently predicted by *lower* CARTS father ratings of sexually abusive behavior. This indicates that, as rated within the CARTS, persons other than biological fathers were more often the perpetrators of sexually abusive behavior in turn associated with high CTQ sexual abuse scores.

**Table 5:** Multiple regression analyses of CARTS convergent validity with Childhood Trauma Questionnaire

		*Step 1: Non-Applicable ratings*	*Step 2: Mother & Father ratings*	*Step 2: Non-Applicable ratings*	*Step 2: Mother ratings*	*Step 2: Father ratings*
		
Dependent measure	Sample	*R*^2^	Δ*R*^2^	*b (SE)*	*b (SE)*	*b (SE)*
CTQ-EA	1	0.22**	0.16**	−1.35 (0.27)**	0.96 (0.45)*	2.17 (0.34)**
	2^a^	0.40**	0.07**	−0.90 (0.16)**	0.45 (0.12)**	0.02 (0.13)
CTQ-PA	1	0.10**	0.16**	−0.02 (0.22)	0.30 (0.40)	2.17 (0.36)**
	2^a^	0.44**	0.07**	−0.70 (0.15)**	0.25 (0.14)	0.47 (0.13)**
CTQ-SA	1	0.26**	0.02*	−1.35 (0.15)**	–	−1.13 (0.45)*
	2^a^	0.57**	0.00	−0.47 (0.05)**	−0.01 (0.24)	0.02 (0.07)

Note: Step-1 predictors were CARTS Non-Applicability ratings whereas Step-2 predictors were CARTS Mother and Father ratings. With dependent measure as CTQ-EA, CTQ-PA, and CTQ-SA, CARTS subscale scores for Emotionally Abusive to Self, Physically Abusive to Self, and Sexually Abusive to Self were used, respectively. CTQ=Childhood Trauma Questionnaire (Bernstein et al., 2003), or screening version (internet sample [2^a^], Thombs et al., 2007); EA=Emotional Abuse; PA=Physical Abuse; SA=Sexual Abuse.

* *p<*0.05, two-tailed

** *p<*0.01, two-tailed.

**Table 6:** Multiple regression analyses of CARTS convergent validity with Lum Emotional Availability of Parents scale

		*Step 1: Non-Applicable ratings*	*Step 2: Mother & Father ratings*	*Step 2: Not Applicable Positive*	*Step 2: Not Applicable Secure*	*Step 2: Parent (M/F) Positive*	*Step 2: Parent (M/F) Secure*
		
Dependent measure	Sample	*R*^2^	Δ*R*^2^	*b (SE)*	*b (SE)*	*b (SE)*	*b (SE)*
LEAP-M	1	0.17**	0.17**	1.50 (0.84)	−2.28 (0.68)*	−0.35 (0.33)	2.63 (0.42)**
LEAP-F	1	0.09**	0.31**	2.24 (1.12)*	−2.10 (0.87)*	1.11 (0.33)**	2.47 (0.47)**

Note: Predictors were CARTS *Positive* and *Secure* subscale scores. Step-2 predictors of LEAP-M were CARTS mother ratings, whereas Step-2 predictors of LEAP-F were CARTS father ratings. LEAP=Lum Emotional Availability of Parents scale (LEAP; Lum & Phares, 2005); LEAP-M=LEAP-Mother; LEAP-F=LEAP-Father.

* *p<*0.05, two-tailed

** *p<*0.01, two-tailed.

Regarding concurrent criterion-related validity, within Samples 1 and 2, CTQ and CARTS ratings together accounted for between 9% (*PANAS-PA;* Sample 2) and 29% (*PANAS-PA;* Sample 2) of the affective outcomes evaluated (see Table 7). In addition, CARTS ratings predicted incremental variance in DASS-Depression, DASS-Stress, and PANAS-NA relative to CTQ emotional, physical, and sexual abuse scores. Increased depressive symptoms were concurrently predicted by CTQ emotional abuse scores in addition to increased CARTS negative feelings from respondents’ fathers, but *decreased* CARTS negative feelings from mothers, and *decreased* CARTS negative beliefs from fathers. Increased stress symptoms were concurrently predicted by CTQ emotional abuse scores, *decreased* CTQ sexual abuse scores, increased CARTS negative feelings from fathers, and *decreased* CARTS negative beliefs from fathers. In comparison, CARTS ratings were not significantly associated with general tendencies toward socially desirable responding or positive impression management as examined within Sample 1 (all τ-b<0.10, *ns*).

**Table 7:** Multiple regression analyses of CARTS incremental and concurrent validity

		*Step 1: CTQ-EA, -PA, -SA*	*Step 2: CARTS Negative Feelings & Beliefs From*	*Step 2 Predictors:*	
		
Dependent Measure	Sample	*R*^2^	Δ*R*^2^	*Subscale*	*b (SE)*
DASS-D	1	0.19**	0.06**	CTQ-EA	0.56 (0.10)**
				CTQ-PA	−0.17 (0.12)
				CTQ-SA	−0.03 (0.16)
				N. Feelings From M	−0.66 (0.24)**
				N. Feelings From F	0.59 (0.23)**
				N. Beliefs From M	0.39 (0.47)
				N. Beliefs From F	−1.04 (0.30)**
DASS-A	1	0.10**	0.02	CTQ-EA	0.31 (0.10)**
				CTQ-PA	−0.07 (0.13)
				CTQ-SA	−0.23 (0.17)
				N. Feelings From M	−0.43 (0.26)
				N. Feelings From F	0.36 (0.24)
				N. Beliefs From M	0.89 (0.51)
				N. Beliefs From F	−0.42 (0.32)
DASS-S	1	0.13*	0.06*	CTQ-EA	0.50 (0.12)*
				CTQ-PA	−0.16 (0.15)
				CTQ-SA	−0.42 (0.20)*
				N. Feelings From M	−0.33 (0.30)
				N. Feelings From F	0.99 (0.29)**
				N. Beliefs From M	−0.12 (0.60)
				N. Beliefs From F	−0.87 (0.38)*
PANAS-NA	2	0.22**	0.07*	CTQ-EA	0.20 (0.10)
				CTQ-PA	0.01 (0.10)
				CTQ-SA	0.08 (0.08)
				N. Feelings From M	0.09 (0.10)
				N. Feelings From F	0.07 (0.09)
				N. Beliefs From M	0.07 (0.09)
				N. Beliefs From F	0.10 (0.08)
PANAS-PA	2	0.03	0.06	CTQ-EA	−0.02 (0.10)
				CTQ-PA	0.10 (0.10)
				CTQ-SA	−0.02 (0.07)
				N. Feelings From M	−0.10 (0.10)
				N. Feelings From F	−0.06 (0.08)
				N. Beliefs From M	0.01 (0.09)
				N. Beliefs From F	−0.12 (0.08)

* *p<*0.05, two-tailed

** *p<*0.01, two-tailed.

### Evaluation of CARTS ratings

#### Paired comparisons between CARTS Mother and Father ratings

Descriptive statistics and paired comparisons between CARTS subscales rated in terms of their descriptiveness for mother vs. father are also reported in Tables 2–4. Across all three samples, mothers were rated as more *Positive* and *Secure* than were fathers. In comparison, across all three samples, fathers were rated as more often the source of self-referential negative feelings, and were rated as being more physically abusive to others in the family. In Samples 2 and 3, fathers were also rated as being more sexually abusive, and respondents indicated that they directed more negative beliefs toward their fathers than toward their mothers. Certain additional differences between how mothers vs. fathers were rated were found to be sample specific (see Tables 2–4).

#### Correlations between CARTS Self, Mother, and Father ratings

Correlations between CARTS subscales rated in terms of their descriptiveness for self vs. mother vs. father are reported in the rightmost columns of Tables 2–4. Across all samples, mother and father ratings exhibited small-to-moderate positive correlations for nearly all CARTS subscales. Associations between endorsements for positively framed items were also consistently correlated among self- and parental-ratings. In comparison, associations between self- and parental-ratings for CARTS items measuring negative affective traits and abusive behavior were somewhat sample specific (see Tables 2–4).

#### Structural equation modeling

Figure 2 shows that, in both student (Fig. 2A, left) and internet samples (Fig. 2B, right), respondents who reported that their father was less emotionally available to them as a child were more likely to self-report experiencing emotional distress during their childhood (*r=*−.27 and *r=*−0.22, respectively, *p*'s<0.001). However, similar associations as referring to mother's emotional availability were significant only within the internet sample (*r=*−0.36, *p*<0.001, vs. *r=*00, *ns*). Further item-level analyses confirmed that mother ratings for none of the emotional availability items correlated with self-ratings for negative affective traits within the student sample, whereas father ratings for all of the same items were significantly correlated.

## Discussion

Instances of childhood abuse and neglect do not occur in a vacuum. Instead, childhood maltreatment typically occurs within the complex social microsystems of families and peer relationships. Despite this fact, standard psychometric approaches to assessing childhood trauma history effectively fail to take account of the socioecological context within which childhood abuse and neglect occurs. What was the relationship between the perpetrator and the victim? How did other family members respond? Who was there to help? Who failed to help? Clinically significant questions such as these are left unanswered by most current psychometric measures of maltreatment history. Indeed current approaches typically assess maltreatment histories in a way that is largely devoid of relational and socioecological context.

We sought to address this concern by developing a new survey methodology and a preliminary set of screening items that we titled the Childhood Attachment and Relational Trauma Screen (CARTS). The CARTS provides a relational-socioecological framework for assessing childhood maltreatment history and the general warmth, security, and supportiveness of the family. Importantly, the CARTS therefore takes account not only of *what* maltreatment may have occurred but also in what relational-socioecological context (i.e., *who* did *what*). Additionally, the CARTS assesses respondents’ own thoughts, feelings, and actions as one way of modeling the general quality of early relationships and what role victims themselves may have played in the co-creation of their family environment.

Across young adult, internet, and outpatient samples, the internal and convergent validity of the CARTS was generally supported, and the inclusion of CARTS ratings specific to parents was often found to incrementally predict variance within conventional measures of childhood trauma and parental emotional availability beyond the general applicability of ratings across family members. These findings demonstrate the incremental utility of assessing histories of childhood abuse and neglect within a socioecological-relational framework. Additionally, evidence for incremental concurrent validity was demonstrated relative to the CTQ (Bernstein et al., 2003), a widely utilized retrospective measure of childhood abuse and neglect. Specifically, it was shown that attributions of parents as the source of negative self-referential feelings and beliefs incrementally predicted concurrent symptoms of depression, stress, and negative affect beyond histories of overt emotional, physical, and sexual abuse (e.g., MacKenzie et al., 2011). Nevertheless, caution is indicated when interpreting the sign of residualized predictors within the multiple regressions wherein associations were sometimes found to be in directions opposite to those predicted.

Most pertinent to our study goals, psychometric support for the CARTS was also proffered in the form of paired tests of mean differences and analyses of association between how different persons were rated (i.e., self vs. mother vs. father). For example, consistent with other studies in North American samples, mothers were considered to be more emotionally available to their children than fathers on average (e.g., Lum & Phares, 2005), and less sexually abusive than fathers. However, mothers were not considered more abusive and neglectful than fathers in general, which is a finding seeming at odds with Canadian national surveys (PHAC, 2010) and therefore requires further research. Results also indicated that warm, secure, and supportive early relationships and caregiving experienced in the context of one's relationship with one's mother and father were correlated, consistent with previous research (e.g., mother and father emotional availability; Lum & Phares, 2005). Moreover, how respondents’ rated themselves in early life was also associated with how they rated their parents in several instances, particularly regarding positively framed items. Finally, SEM illustrated the ability of CARTS relational-socioecological contextualized data to represent associations of theoretical interest to the study of childhood abuse and neglect and parental attachment in more complex ways. Specifically, it was shown that self-reported childhood negative-affectivity was more likely in the presence of rated parental lack of *emotional availability* (Fig. 2). However, within undergraduates, only paternal emotional availability was associated with less trait negative affect during childhood, while maternal emotional availability was unrelated to childhood negative affect. This surprising result, although requiring replication, if nothing else draws attention to the otherwise often overlooked influence of paternal attachment figures in the development of emotional behavior within their offspring (e.g., Phares, 1992; Phares, Fields, Kamboukos, & Lopez, 2005).

We acknowledge the limitations of our work. Firstly, we recognize that additional psychometric work on the CARTS item set will be needed. For example, we regarded the present sample sizes as insufficient to merit empirical analyses of the rationally derived subscale structure, and the adequacy of the content representation of the item set requires further investigation. Secondly, the representativeness of the current samples may be in question; for example, women were overrepresented, completers of the internet survey differed from non-completers on a number of demographic variables, the diagnostic status of the mental health outpatient sample was not systematically assessed, there was relatively little endorsement of emotional, physical, or sexual abuse within the undergraduate sample, and participants who did not report on both biological parents were not analyzed. Future studies are therefore necessary to determine the generalizability of our results across populations differing by gender distribution, demographic and sociocultural characteristics, mental health status, extent of childhood maltreatment history, and family constitution. Thirdly, future researchers should investigate the susceptibility of CARTS ratings to variability in mood state at the time of testing such as through test–retest studies and experimental mood-state inductions. Fourthly, the time period retrospectively measured (childhood and adolescence), although intended to match that used by the CTQ and many other standard maltreatment inventories, was nevertheless perhaps too broad—for example, certain participants noted during debriefing that their relationship with their parents changed significantly when they entered adolescence. Therefore, future studies may wish to modify the instructional set such that the time period investigated is narrower, perhaps referring to distinct developmental periods (e.g., early childhood only, or adolescence only). Fifthly, the present study limited its analyses to responses concerning the self, mother, and father only, and only few items were considered relevant to description of the respondent him or herself; it will likely be informative for future researchers to evaluate relationships with other family members (e.g., siblings, grandparents, etc.) and vary the extent to which the item set is self-referential in nature based on specific study objectives. Moreover, the family listing provided by participants may be insufficiently inclusive as a descriptive of participants’ families, and limiting maltreatment assessment to family members precludes endorsement of abuse perpetrated by persons outside the family; future studies should assess the effects of prompting participants to include additional family members as well as making possible the endorsement of items as referring to persons other than those previously listed as family members. Sixthly, the current items do not provide for a detailed, behaviorally explicit assessment of trauma exposure. Seventhly, the choice of stick-figure icons, being that they are not gender neutral, may be considered offensive to some participants. Finally, we again emphasize that abusive and neglectful experiences that may have occurred outside the family structure were not examined by the CARTS; future studies might modify the current methodology so as to be more inclusive, including ways to measure not only the family microsystem but also the influence of exosystems (broad communities) and macrosystems (society, culture).

Our preliminary evidence suggests the promise of the CARTS as a new survey methodology for assessing the warmth, security, and supportiveness of early attachment relationships and the occurrence of relational trauma. The procedure makes possible a relationally and socioecologically informed assessment framework. Future research addressing the limitations of the present studies is indicated.
